# Radiological age assessment based on clavicle ossification in CT: enhanced accuracy through deep learning

**DOI:** 10.1007/s00414-024-03167-6

**Published:** 2024-01-30

**Authors:** Philipp Wesp, Balthasar Maria Schachtner, Katharina Jeblick, Johanna Topalis, Marvin Weber, Florian Fischer, Randolph Penning, Jens Ricke, Michael Ingrisch, Bastian Oliver Sabel

**Affiliations:** 1 Department of Radiology, LMU University Hospital, LMU Munich, Marchioninistraße 15, 81377 Munich, Germany; 2 Munich Center for Machine Learning (MCML), Geschwister-Scholl-Platz 1, 80539 Munich, Germany; 3 Comprehensive Pneumology Center (CPC-M), Member of the German Center for Lung Research (DZL), Munich, Max-Lebsche-Platz 31, 81377 Munich, Germany; 4 Institute of Informatics, LMU Munich, Oettingenstraße 67, 80538 Munich, Germany; 5 Institute of Forensic Medicine, LMU Munich, Nußbaumstraße 26, 80336 Munich, Germany

**Keywords:** X-Ray computed tomography, Age determination by skeleton, Deep learning, Sternoclavicular joint, Forensic medicine

## Abstract

**Background:**

Radiological age assessment using reference studies is inherently limited in accuracy due to a finite number of assignable skeletal maturation stages. To overcome this limitation, we present a deep learning approach for continuous age assessment based on clavicle ossification in computed tomography (CT).

**Methods:**

Thoracic CT scans were retrospectively collected from the picture archiving and communication system. Individuals aged 15.0 to 30.0 years examined in routine clinical practice were included. All scans were automatically cropped around the medial clavicular epiphyseal cartilages. A deep learning model was trained to predict a person’s chronological age based on these scans. Performance was evaluated using mean absolute error (MAE). Model performance was compared to an optimistic human reader performance estimate for an established reference study method.

**Results:**

The deep learning model was trained on 4,400 scans of 1,935 patients (training set: mean age = 24.2 years ± 4.0, 1132 female) and evaluated on 300 scans of 300 patients with a balanced age and sex distribution (test set: mean age = 22.5 years ± 4.4, 150 female). Model MAE was 1.65 years, and the highest absolute error was 6.40 years for females and 7.32 years for males. However, performance could be attributed to norm-variants or pathologic disorders. Human reader estimate MAE was 1.84 years and the highest absolute error was 3.40 years for females and 3.78 years for males.

**Conclusions:**

We present a deep learning approach for continuous age predictions using CT volumes highlighting the medial clavicular epiphyseal cartilage with performance comparable to the human reader estimate.

**Supplementary Information:**

The online version contains supplementary material available at 10.1007/s00414-024-03167-6.

## Background

Radiological age assessment is a method that examines certain physiological properties in radiographic or computed tomography (CT) images to estimate a person’s chronological age [1, 2]. In this study, we explore a potential approach to enhance radiological age assessment based on clavicle bone ossification through deep learning.

### Importance of age

In many countries, age governs the relationship between individuals and the state. Changes in age can lead to the acquisition of rights and obligations, such as emancipation, employment, criminal responsibility, sexual relation, consent for marriage, or military service [3]. Thus, age is a critical component of a person’s identity, particularly for children. The United Nations Convention on the Rights of the Child (CRC, Article 1) [4] and the EU acquis (Directive 2013/33/EU, Article 2(d)) [5] define a child as any person below the age of 18. States and authorities have specific age-related obligations under the CRC that include: registration of the child after birth, respecting the right of the child to preserve his or her identity, and speedily re-establish his or her identity in the case that some or all elements of the child’s identity have been deprived [3]. In cases where a person’s age is unknown or in serious doubt, a state may need to assess the age, e.g., to determine whether they are an adult or a child. The European Union Agency for Asylum (EUAA) recommends using the least intrusive age assessment method possible, gradually implementing more invasive methods if necessary, and selecting the most accurate method while documenting the margin of error [3]. Radiological age assessment is one such method and its accuracy may be improved using deep learning. Other non-binding recommendations from local expert panels exist, e.g., from the Working Group for Forensic Age Diagnostics of the German Society for Forensic Medicine (AGFAD).^1^

### Reference study-based radiological age assessment

Radiological age assessment is based on examinations of body parts that capture the skeletal development of the person whose age is unknown, such as the carpal bones, the molars, or the clavicles [1]. In this study, we focus on the ossification status of the medial clavicular epiphyseal cartilages, as they are the last maturing bone structures in the human body, and enable the estimation of a wide range of ages, from teenagers to young adolescents and adults [6]. Typically, atlas methods [2] or reference study methods [7–9] are applied for age assessment, where the age of the examined person is assumed to be similar to the reference person or case group with similar skeletal maturation.

However, these methods have several limitations. First, the number of case groups is finite, e.g. n = 9 in [7–9], which limits the accuracy of age estimates. Second, age differences between members of the same case group can be large, e.g., up to 14.2 years [7], leading to high uncertainties. Third, expanding control groups is challenging because the assessment of the ossification stage by experts is time-consuming. Finally, these methods are subject to intra- and inter-reader variability [10, 11].

### Deep learning-based radiological age assessment

A promising tool for more accurate radiological age assessment via the clavicle bones is deep learning. It has been successfully applied in a variety of computer vision tasks in medical imaging [12] including radiological age assessment through dental radiographs [13], knee MRIs [14], and more [15]. The large amounts of data required to train a deep network for age assessment [16]—medical images including clavicles and sternum, along with the corresponding age information—are abundant in many hospitals and can be accessed retrospectively through their picture archiving and communication systems (PACS). Furthermore, data from institutions in different locations can be combined to form a dataset that is representative of the global population as well as possible. Finally, feed-forward deep learning models are deterministic as the same input image always results in the same output and age predictions do not suffer from intra- or inter-rater variability. This might be an advantage when considering which method should be deployed in potential legal scenarios.

Therefore, we (a) propose a deep learning approach to predict the chronological age based on CT image volumes of the medial clavicular epiphyseal cartilage and (b) compare it to a favorable human reader performance estimate for the reference study method of Kellinghaus et al. [7, 8]. It is widely acknowledged in conventional practice that the classification of stage 3b in males and stage 3c in females following the Kellinghaus method suggests a minimum age of 18 years or above.

## Methods

### Retrospective data collection

**Fig. 1:**
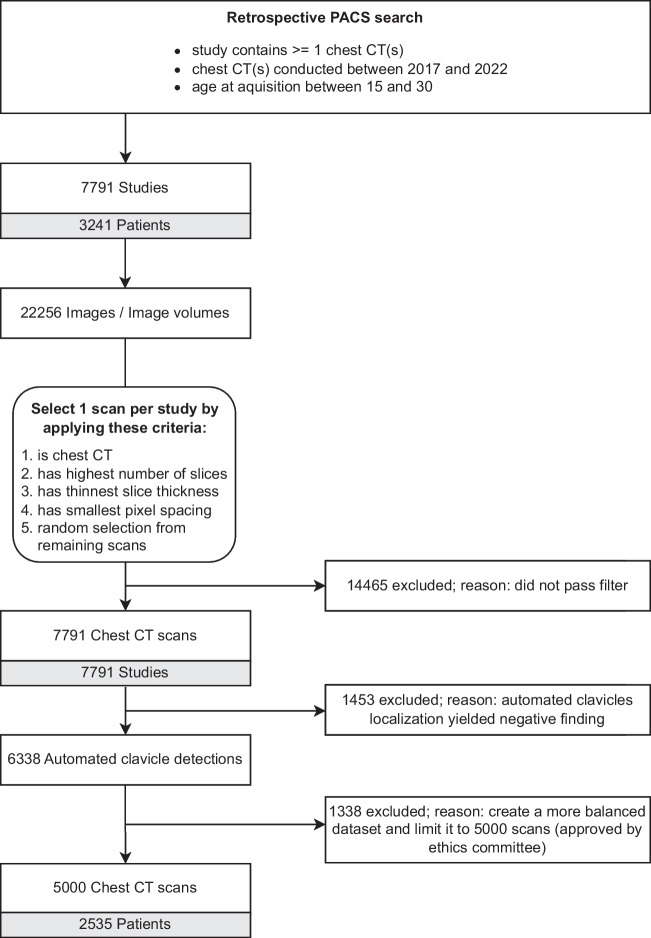
CT scan inclusion diagram. Flow diagram of the selection process from study identification in the picture archiving and communication system (PACS) to the chest CT scans in the dataset

This retrospective study was approved by the institutional review board (Ethics Committee, Medical Faculty, LMU Munich) and the requirement for written informed consent was waived. CT scans were collected retrospectively from the PACS of LMU Munich’s University Hospital. We specifically searched for chest CT scans of persons between the ages of 15.0 and 30.0 years, with documented sex, reimbursed by a recognized health-insurance provider (state-mandated or private), acquired during the clinical routine for all purposes between 2017 to 2020. To ensure truthful age information we excluded scans issued and paid for by state agencies, which among other things excludes requests for forensic age assessments. Age was calculated as the number of days between the date of birth and the date of examination. The selected age range covers a broad spectrum of skeletal developmental stages of the medial clavicular epiphyseal cartilages [17]. One scan per study was selected based on multiple criteria specified in the flow diagram in Fig. 1, which summarizes the entire data collection process.

### Deep learning model

**Fig. 2:**
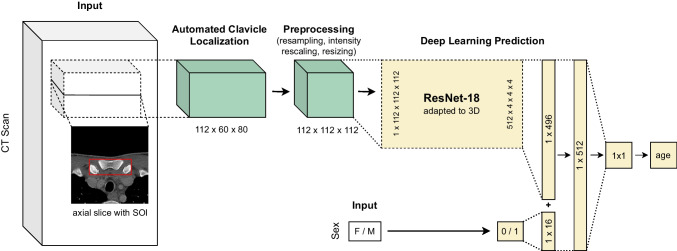
Deep learning-based radiological age assessment. Schematic visualization of the proposed approach for deep learning-based radiological age assessment. First, the CT scan is cropped around the automatically localized structures of interest (SOIs), which are the medial clavicular epiphyseal cartilages. Second, the scan undergoes several preprocessing steps which include resampling, intensity rescaling, and resizing. Finally, the adapted three-dimensional ResNet-18 predicts chronological age based on the preprocessed scan. Additionally, sex information is incorporated into the approach by fusing it with the image embedding before the last fully connected layer. While the figure only depicts a single network, the deep learning approach uses a deep ensemble consisting of 20 uniquely trained networks

A schematic overview of the deep learning approach for radiological age assessment is shown in Fig. 2. We express age assessment as a regression analysis where the dependent variable (age) is a scalar, which is estimated based on a feature (CT scan), by a deep learning model. The model in this study was an ensemble [18] of 20 deep neural networks (deep ensemble) that share the same architecture and training process. The mean of the predictions from the 20 ensemble members was used as the ensemble prediction. The architecture was adapted from the popular ResNet-18 [19], where we replaced the two-dimensional convolutions with three-dimensional convolutions to enable processing CT volume inputs, and added a second input to process sex information.

Prior to model training, the collected CT scans were preprocessed (described in detail in the supplement) including an automated localization of the clavicles [20]. This localization also served as a filter for chest CT scans that do not include the clavicles or scans wrongly labelled as chest CT. Next, the dataset was split into a training, a validation, and a test set. Validation and test set were sampled to include not more than one CT scan of the same person and to have the same equal number of samples per age (bin size = 1 year) and sex. All remaining samples from persons not in the validation or test set were used as the training set. No person is part of more than one set. The deep ensemble was trained on the training set, and training progress was monitored using the validation set. Model performance was evaluated by measuring the absolute error of model predictions for the test set. Details regarding the dataset split, model, and training are provided in the supplement.

### Abstention-performance trade-off

We applied the estimated predictive uncertainty of the deep ensemble to identify samples with a potentially high prediction error. The standard deviation (SD) of the predictions made by the ensemble members for a given input served as the respective uncertainty estimate [21]. In an abstention-performance trade-off, we abstain from predictions for the fraction of samples with the highest measured uncertainties (abstention rate) to improve average performance for the remaining samples. For example, in a trade-off with an abstention rate of 20%, we rank all predictions by predictive uncertainty and analyze only the top 80% of samples with the lowest uncertainty. This allows the machine learning model to say “I don’t know” [22] in cases where it is unsure, instead of forcing an answer at all costs.

### Optimistic human reader performance estimate

To classify the performance of our deep learning model, we calculated an optimistic human reader performance estimate for the radiological age assessment of Kellinghaus et al. [7, 8]. This method is based on 9 clavicle ossification stages, with three major stages (1, 4, and 5) and 6 substages (2a—2c and 3a—3c). They range from no ossification of the ossification center (stage 1) to complete fusion of the epiphyseal cartilage (stage 5). An individual’s age is estimated by first determining the ossification stage in a radiological examination [7, 8]. Next, the age is derived from the age distribution of a case group of known age and with the same ossification stage and sex.

**Fig. 3:**
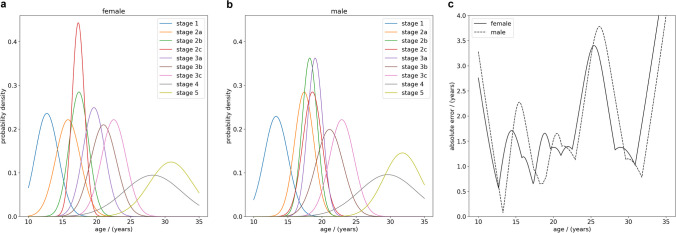
Optimistic human reader performance estimate. The left and center panels display the probability density of a person being in a certain ossification stage, based on normal distributions described in [7, 8], for (**a**) females and (**b**) males between the ages of 10 and 35 years. The right panel (**c**) shows the best-case mean absolute error estimate of predicted ages for true ages between 10 and 35 years when applying the radiological reference study method for age assessment of Kellinghaus et al. [7, 8]

The human reader estimate assumes a best-case scenario in which (a) the descriptive ossification stage statistics described in [7, 8] are derived from a cohort that is representative of all individuals, in particular, our test set, (b) age in each stage follows a normal distribution and (c) trained reviewers always assess the correct ossification stage. Under these conditions the HRE provides the lower limit for the absolute error that can be achieved with the reference study method when applied to a person with a certain true age $x$ (Fig. 3).

For a given age $x$ we first calculated the absolute difference to the mean age $M$ of each ossification stage $s$:

$$| x- M(s) |$$

For example, for a 21.00 year old male, these differences are 7.72 years for stage 1 (M = 13.28 years), 3.60 years for stage 2a (M = 17.40 years), 2.80 years for stage 2b (M = 18.20 years), 2.40 years for stage 2c (M = 18.60 years), 2.00 years for stage 3a (M = 19.00 years), 0.10 years for stage 3b (M = 21.10 years), 1.90 years for stage 3c (M = 22.90 years), 8.63 years for stage 4 (M = 29.63 years), 10.77 years for stage 5 (M = 31.77 years).

Next, we calculated the probability density ${p}_{s}(x)$ (Fig. 3) for a person with the true chronological age $x$ to be in ossification stage $s$ based on normal distributions calculated from the provided mean and SD values. The probabilities were normalized such that

$$\sum\limits_{s=1}{p}_{s}(x)=1 .$$

It is important to note, that two persons of the same chronological age can be in two different ossification stages. In the example of a 21.00 year old male, these probabilities are ${p}_{1}=2.45 \times {10}^{-5}$, ${p}_{2a}=2.10 \times {10}^{-2}$, ${p}_{2b}=2.86 \times {10}^{-2}$, ${p}_{2c}=1.32 \times {10}^{-1}$, ${p}_{3a}=1.40 \times {10}^{-1}$, ${p}_{3b}=4.01 \times {10}^{-1}$, ${p}_{3c}=2.55\times {10}^{-1}$, ${p}_{4}=2.24\times {10}^{-2}$, and ${p}_{5}=1.29\times {10}^{-4}$.

The probability densities ${p}_{s}(x)$ were multiplied by the absolute difference to the mean age.

$${p}_{s}(x) \cdot | x- M(s) |$$

The sum of these products for all ossification stages yielded the absolute error of the reference study method for a person with the true age $x$:

$$AE (x) =\sum_{s=1}\left|M\left(s\right)-x\right|{p}_{s}\left(x\right)$$

In the example of the 21.00 year old male, the AE is 1.64 years. The MAE of the reference study method for all individuals in the test set was then given by:

$$MAE=\frac{1}{\left|{X}_{Test}\right|}{\sum }_{x \in {X}_{Test}}AE\left(x\right).$$

### Classical expert reader age assessment

A senior radiologist and expert in the field conducted a manual reading of a small subset of the test set, comprising 50 randomly sampled test set scans. The reading followed the Kellinghaus method [7, 8] and assessed the ossification stages 1, 2a, 2b, 2c, 3a, 3b, 3c, 4, and 5. The mean age value of each stage of the respective sex was used as age prediction for the manual reading.

## Results

### Dataset

A retrospective search in our hospital’s PACS identified 7,791 studies conducted between 2017 and 2020 on 3,241 patients that involved at least one chest CT scan with a recorded age at acquisition between 15 and 30 years, documented sex, and recognized health insurance provider (state-mandated or private). The 7,791 studies included 22,256 images or image volumes. Some studies included more than one chest CT scan that would have been suitable for analysis. After scan selection (Fig. 1), the final dataset consisted of 5,000 chest CT scans from 2,535 patients (mean age = 24.2 ± 4.0 years), with 44% (1,103/2,535) females. The training set consisted of 4,400 scans from 1,935 patients, with 41% (803/1,935) female. The validation and test set were independent and both included 300 scans from 300 patients (both: mean age = 22.5 ± 4.4 years), 10 scans per age (bin size = 1 year), and sex, with 50% (150/300) being female. All datasets are summarized in Table 1 and their age distribution is shown in supplementary Figure S3.

**Table 1:** Documentation of the number of patients and CT scans in the total dataset, as well as in the training, validation, and test set

Set	**Total**		**Training**	
Patients	f	m	f	m
	1103 (44%)	1432 (56%)	803 (41%)	1132 (59%)
	2535		1935	
CT scans	5000		4400	
Set	**Validation**		**Test**	
Patients	f	m	f	m
	150 (50%)	150 (50%)	150 (50%)	150 (50%)
	300		300	
CT scans	300		300	

### Deep learning-based radiological age assessment

**Fig. 4:**
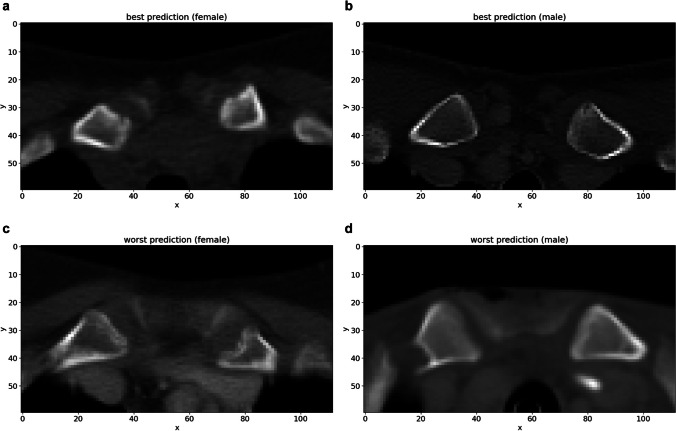
Test set input examples. Selected axial slices of the preprocessed CT scans of (**a**) the best female, (**b**) best male, (**c**) worst female, and (**d**) worst male deep learning prediction for age. The worst predictions show (**c**) a “fish mouth configuration of the left clavicle” and a (**d**) osteolytic lesion of the right clavicle

**Fig. 5:**
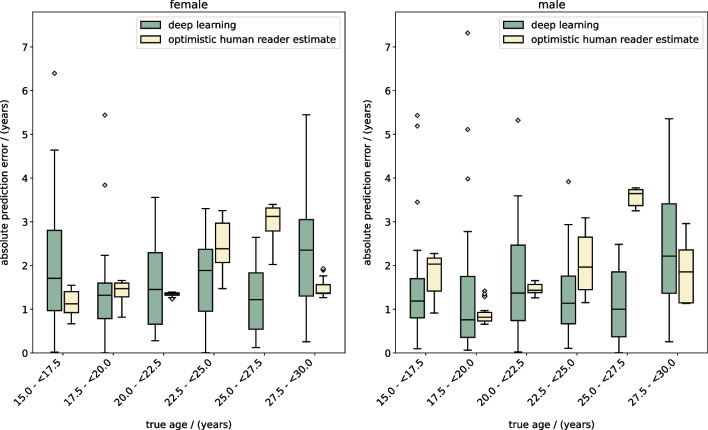
Radiological age assessment results. Absolute prediction error of the (green) deep learning approach and the (yellow) optimistic human reader performance estimate for radiological age assessment of (left panel) females and (right panel) males between 15 and 30 years. The boxes extend from the lower to the upper quartile age values of each bin, with a line at the median. The whiskers extend from the boxes to 1.5 × interquartile range (IQR) (Q3—Q1) in each direction. Flier points are age values past the end of the whiskers

The deep ensemble model (Fig. 2) was trained using the training data and training was monitored using the validation data. The model's performance was evaluated on the test data. The results showed a mean absolute error (MAE) of 1.65 years (standard deviation (SD) = 0.53) for all patients, 1.69 years (SD = 0.53) for female patients, and 1.62 years (SD = 0.54) for male patients. The best prediction for a female individual had an absolute error of 0.003 years (true age = 18.604 years), while the best prediction for a male had an absolute error of 0.005 years (true age = 25.142 years). The corresponding input CT scans are displayed in Fig. 4 and show no medical abnormalities. The worst prediction for a female had an absolute error of 6.40 years (true age = 15.29 years). The corresponding CT showed a fish mouth shape variant with concavely configured clavicle ends in the left clavicle (Fig. 4). Shape variants near the sternal ends of the clavicle occur frequently and severely limit assessability [23, 24]. The worst prediction for a male had an absolute error of 7.32 years (true age = 19.20 years). A CT examination revealed that the individual had osteolysis in the right clavicle, presumably as a manifestation of an underlying malignant disease (Fig. 4). The distribution of absolute errors by age (bin width = 2.5 years) is shown separately for male and female patients in Fig. 5. The MAE, maximum absolute error (max error), and the 90th percentile absolute error (p90 error) for each age (bin width = 1 year) are reported in Table 2 for female individuals and Table 3 for male individuals.

**Table 2:** Radiological age assessment results for female persons in the test set using deep learning (DL) and optimistic human reader performance estimate (HRE). The table displays the mean absolute error (MAE), maximum absolute error (max error), and 90th percentile absolute error (p90 error) in years. The number of individuals in each age group was n = 10

Results for female subjects						
Age	Deep learning			Human reader estimate		
	MAE	Max err	p90 err	MAE	Max err	p90 err
15.0- < 16.0	2.85	6.40	4.82	1.41	1.55	1.51
16.0- < 17.0	1.68	2.90	2.78	1.02	1.15	1.14
17.0- < 18.0	1.16	3.84	1.80	0.90	1.23	1.13
18.0- < 19.0	0.94	1.88	1.76	1.57	1.66	1.66
19.0- < 20.0	1.70	5.44	2.55	1.45	1.55	1.55
20.0- < 21.0	1.61	2.48	2.44	1.33	1.38	1.38
21.0- < 22.0	1.18	3.56	2.42	1.34	1.39	1.39
22.0- < 23.0	2.01	3.52	3.14	1.49	1.73	1.70
23.0- < 24.0	1.52	3.30	2.92	2.24	2.56	2.52
24.0- < 25.0	1.72	2.61	2.43	3.02	3.26	3.25
25.0- < 26.0	1.24	2.64	2.36	3.34	3.40	3.39
26.0- < 27.0	1.15	2.03	1.94	2.88	3.20	3.06
27.0- < 28.0	1.45	3.05	2.98	1.85	2.35	2.06
28.0- < 29.0	2.55	5.45	4.21	1.37	1.39	1.38
29.0- < 30.0	2.53	4.24	3.78	1.34	1.37	1.37

**Table 3:** Radiological age assessment results for male persons in the test set using deep learning (DL) and optimistic human reader performance estimate (HRE). The table displays the mean absolute error (MAE), maximum absolute error (max error), and 90th percentile absolute error (p90 error) in years. The number of individuals in each age group was n = 10

Results for male subjects						
Age	Deep learning			Human reader estimate		
	MAE	Max err	p90 err	MAE	Max err	p90 err
15.0- < 16.0	1.29	2.35	1.85	2.18	2.27	2.27
16.0- < 17.0	1.74	5.43	3.65	1.7	2.06	2.04
17.0- < 18.0	1.1	5.19	2.08	0.9	1.21	0.99
18.0- < 19.0	1.26	5.11	2.53	0.7	0.77	0.75
19.0- < 20.0	2.18	7.32	4.32	1.12	1.42	1.36
20.0- < 21.0	1.44	3.04	2.42	1.6	1.65	1.65
21.0- < 22.0	1.86	3.35	3.34	1.42	1.47	1.45
22.0- < 23.0	1.72	5.32	3.77	1.25	1.32	1.31
23.0- < 24.0	1.34	3.92	2.76	1.66	2.14	1.99
24.0- < 25.0	1.48	2.94	2.82	2.69	3.1	3.01
25.0- < 26.0	0.96	2.26	1.91	3.54	3.76	3.7
26.0- < 27.0	1.11	2.43	1.91	3.68	3.78	3.78
27.0- < 28.0	1.57	3.37	2.57	2.97	3.33	3.32
28.0- < 29.0	2.57	4.75	3.97	2.01	2.39	2.28
29.0- < 30.0	2.67	5.36	4.48	1.19	1.51	1.24

### Optimistic human reader radiological age assessment

The human reader estimate for the radiological age assessment method of Kellinghaus et al. [7, 8] was applied to the test set. The results showed a MAE of 1.84 years (SD = 0.84 years) overall, 1.77 years (SD = 0.74 years) for female individuals, and 1.91 years (SD = 0.92 years) for male individuals. The distribution of absolute errors by age (bin width = 2.5 years) is shown separately for male and female individuals in Fig. 5. The MAE, max error, and p90 error for each age (bin width = 1 year) are reported in Table 2 for female patients and Table 3 for male patients.

### Classical expert reader age assessment

The manual age assessment of 50 randomly sampled test set scans by an expert in the field following the method of Kellinghaus et al. [7, 8] yielded a MAE of 1.97 years (SD = 1.48 years). For comparison, the deep learning model achieved a MAE of 1.44 years (SD = 0.95 years) on the same subset.

### Abstention-performance trade-off

**Fig. 6:**
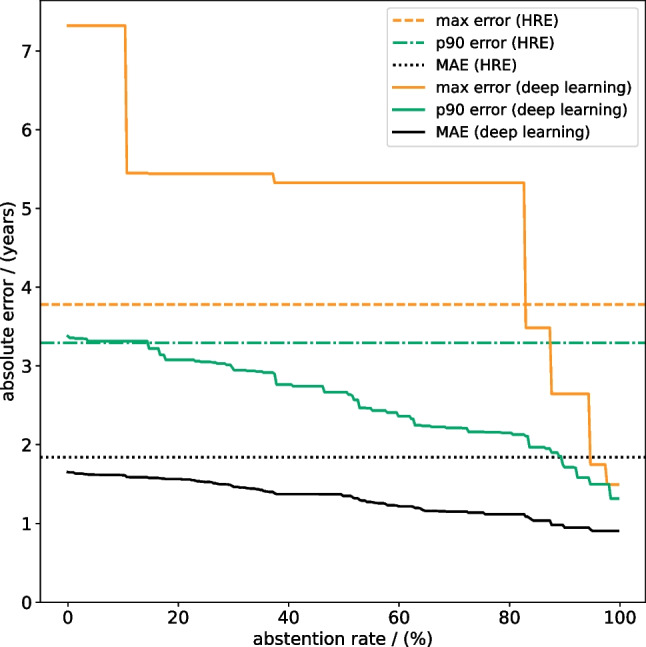
Abstention-performance trade-off. The abstention-performance trade-off for deep learning-based radiological age assessment, where we abstain from analysis for predictions with the highest predictive uncertainties. Optimistic human reader performance estimate (HRE) results are included for reference. Increasing abstention rates lead to an improved deep learning mean absolute error (MAE), maximum absolute error (max error), and 90th percentile absolute error (p90 error). For abstention rates > 14.7% the p90 error of the deep learning model is below 3.22 years and better compared to the human reader estimate (p90 error = 3.29 years). For abstention rates > 82.9% the max error of the deep learning model is below 3.49 years and better compared to the human reader estimate (max error = 3.78 years)

In a separate analysis, we applied an abstention-performance trade-off to the deep learning model predictions, i.e. we did not take results from samples with the highest predictive uncertainties into account. MAE, max error, and p90 error for abstention rates ranging from 0% (all samples evaluated, no abstention) to 100% (no samples evaluated) are shown in Fig. 6. All metrics decreased for increasing abstention rates, i.e. the greater the fraction of the most uncertain predictions that were not considered for analysis, the better the remaining predictions on average. For abstention rates > 14.7% the p90 error of the deep learning model was below 3.22 years and outperformed the human reader estimate which had a p90 error of 3.29 years. For abstention rates > 82.9% the max error of the deep learning model was below 3.49 years and thus better compared to the human reader estimate which had a max error of 3.78 years. Table 4 reports the deep learning model’s and human reader estimate’s MAE, max error, and p90 error separately for female and male individuals, and for abstention rates of 20% and 50%.

**Table 4:** Summary of radiological age assessment results for the test set using deep learning (DL) and optimistic human reader performance estimate (HRE). The table displays the mean absolute error (MAE), absolute error standard deviation (SD), maximum absolute error (max error), and 90th percentile absolute error (p90 error) for female and male persons. Additionally, the table shows the deep learning results with an abstention-performance trade-off for abstention rates of 20% and 50%, where predictions with the highest predictive uncertainty (20 or 50% of predictions) are not taken into account for analysis

	MAE (SD)		max error		p90 error	
	f	m	f	m	f	m
HRE	1.84 (0.84)		3.40	3.78	3.29	
	1.77 (0.74)	1.91 (0.92)			3.08	3.42
DL	1.65 (1.27)		6.40	7.32	3.38	
	1.69 (1.18)	1.62 (1.34)			3.10	3.45
DL (20% AR)	1.57 (1.14)		5.44	5.32	3.12	
	1.61 (1.09)	1.52 (1.18)			3.06	3.36
DL (50% AR)	1.35 (1.00)		4.24	5.32	2.67	
	1.44 (0.99)	1.26 (1.00)			2.91	2.54

## Discussion

Radiological age assessment methods based on reference studies analyzing the ossification of the sterno-clavicular joint are inherently limited in accuracy due to their design. The clavicles specifically enable age assessment for older minors (15–18 years), adolescents (18–21 years), and young adults (21–30 years). In an optimistic human reader performance estimate, we calculated that the well-established method of Kellinghaus et al. [7–9] cannot predict chronological age more accurately than 1.84 years on average and no better than 0.66 years at best for individuals whose true age is between 15.0 and 30.0 years.

### Deep learning model

In an effort to overcome this inherent limitation, we developed a deep learning approach for radiological age assessment based on clavicle ossification (Fig. 2). The deep learning model outperformed the human reader estimate of the Kellinghaus et al. method on average and achieved a MAE of 1.65 years on a balanced test dataset containing 300 chest CT volumes that have been cropped around the sterno-clavicular joints.

While the superior average performance highlights the potential of deep learning, ensuring the algorithm’s safety for all individuals is crucial. Consequently, the model’s highest error should be low and high errors should be infrequent during testing. Deep learning returned absolute errors up to 7.32 years and fell short of the human reader estimate which only had absolute errors up to 3.78 years (Table 4). However, the samples that returned the worst deep learning predictions showed norm-variants or pathologic disorders, which would be exclusion criteria for radiological age assessment with reference study methods [23, 24].

Additionally, rare high error predictions can be avoided for deep learning with an abstention-performance trade-off (Fig. 6): we leveraged predictive uncertainty to identify potential high error predictions, excluded them from analysis, and improved performance for the remaining predictions. For abstention rates > 14.7% the deep learning model surpassed the human reader estimate’s p90 error of 3.29 years, indicating the potential for reducing high errors during application.

Another benefit of automated deep learning age assessment is the significantly reduced analysis time for scans. This advantage may valuable in post-mortem CT examinations for identification purposes, e.g. following mass casualty incidents.

### Positioning within the literature

To the best of our knowledge, no deep learning-based age assessment using chest CT volumes of the clavicles has been reported yet. However, several pioneering studies leverage other imaging modalities to predict age based on different skeleton areas. Auf der Mauer et al. [14] analyzed 185 coronal and 404 sagittal 3D knee MRI volumes of Caucasian male subjects between the age of 13.0 and 21.8 years and middle to high socio-economic status. Using a combination of a deep learning model and a classical decision tree-based machine learning algorithm, they could improve the MAE from 1.63 (SD = 0.99) years achieved by a naive baseline model, which always predicts the mean age of the training set, to 0.69 (SD = 0.49) years. Vila-Blanco et al. [13] studied 2,289 2D dental panoramic radiograph images of Spanish Caucasian subjects in the age range of 4.5 to 89.2 years. Their deep learning model achieved an MAE of ~ 2.5 years for the subgroup of 798 subjects between the ages of 15.0 and 30.0 years. In the 2017 RSNA Pediatric Bone Age Machine Learning Challenge [25], participants trained deep learning models to predict expert-assigned bone age.

### Limitations

This study has limitations. First, the complex relationship between skeletal development and chronological age poses an insurmountable natural accuracy barrier [26] for age assessment and depends on a variety of factors ranging from genetic predisposition to socio-economic status [27]. Second, the data used to train, validate, and test the deep learning model was collected retrospectively and acquired during the clinical routine for all purposes. Therefore, it was inhomogeneous, acquired with different scanners using different protocols, and includes samples that would have been ruled out for radiological age estimation by experts based on the health condition of the individual. Third, all CT scans in our dataset were acquired at the same hospital, which likely introduced a bias that prevents the data from being representative of the global population. Fourth, the training dataset included multiple CT scans per individual (4400 CT scans vs. 1935 individuals), while only one unique scan per individual was used in the validation and test dataset (300 CT scans and 300 individuals, respectively). Additionally, the dataset included only CT scans for which the automated localization of the medial clavicular epiphyseal cartilages returned a positive detection. Finally, the human reader estimate is based on the statistics reported by Kellinghaus et al. [7, 8], but other studies applying the same method exist, e.g. from Wittschieber et al. [9].

### Ethics disclaimer

We do not endorse the actual or exploratory application of the approach presented in this study for radiological age assessment. Instead, we suggest further research into deep learning approaches for radiological age assessment under controlled settings, following the promising results in this and similar studies. Specifically, we recommend transferring the presented approach from CT to magnetic resonance imagining (MRI) data to avoid exposing individuals to potentially harmful ionizing radiation. The MRI dataset should be extensive and inclusive to ensure its representation of all individuals. The research should also focus on reducing deep learning prediction variance and extreme errors.

### Conclusion

In summary, our study demonstrates a deep learning approach for radiological age assessment using CT volumes that highlight the medial clavicular epiphyseal cartilages. Deep learning surpassed the human reader performance estimate in terms of mean accuracy (MAE = 1.65 vs. 1.84 years). Errors could partially be attributed to physiological abnormalities. Also, high errors may be avoided by abstaining from predictions with high uncertainty. Looking ahead, deep learning offers an accurate, objective, and scalable solution that eliminates intra- and inter-reader variability and could be further improved with larger and standardized datasets.

### Supplementary Information

Below is the link to the electronic supplementary material.

Supplementary file1 (DOCX 365 kb)

## Data Availability

The datasets generated and analyzed during the current study are not publicly available due to them containing information that could compromise research participant privacy, but are available from the corresponding author on reasonable request and with permission of the institutional review board (Ethics Committee, Medical Faculty, LMU Munich).

## Footnotes

1 https://www.dgrm.de/forensische-altersdiagnostik/empfehlungen (2023/11/28).

## Code availability

The underlying code for this work is available in *Github* and can be accessed via this link https://github.com/pwesp/dl-rad-age.
