# Supplementary material for: Radiological age assessment based on clavicle ossification in CT: enhanced accuracy through deep learning
Source: Int J Legal Med. 2024 Jan 30;138(4):1497–507. doi: 10.1007/s00414-024-03167-6 (PMC11164764; doi:10.1007/s00414-024-03167-6)
Supplement: Supplementary file 1 — Supplementary file1 (DOCX 365 kb) [file 414_2024_3167_MOESM1_ESM.docx]

# Supplementary information

## Scan selection

This section describes the scan selection process we applied to the 7,791 studies including 22,256 images or image volumes retrospectively acquired from the PACS in order to get the final dataset. Some studies included more than one chest CT scan that would have been suitable for analysis. To avoid study duplicates and minimize patient duplicates, a single chest CT scan was selected from each study by applying the following criteria in order: (a) chest CT, (b) highest number of slices, (c) thinnest slice thickness, (d) minimal pixel spacing, and (e) random selection from remaining scans. Because the skeletal structures of interest are the sterno-clavicular joints, we applied an automated clavicle detection algorithm [[20]](https://www.zotero.org/google-docs/?Mc5CCl) to each scan, which returned a positive detection in 6,338/7,791 (81.3 %) of the original scans. Also, the number of scans increased with age at acquisition. To create a more balanced dataset and limit it to 5,000 scans (approved by the ethics committee), we removed 1,338/6,338 (21%) scans in an iterative process. In each step, we randomly removed a scan of a patient with the most frequent age (bin size = 1 year), and with more than one scan in the dataset, if possible. After scan selection, the final dataset consisted of 5,000 chest CT scans from 2,535 patients, with 44% (1,103/2,535) females (Figure S3). The scan selection is summarized in the CT scan inclusion diagram in Figure 1.

## Raw data properties

The 5,000 selected chest CT scans had a mean pixel spacing of 0.82 x 0.82 mm^2^ (SD = 0.19 x 0.19 mm^2^) and a mean slice thickness of 2.60 mm (SD = 1.44 mm). The distribution of pixel spacings and slice thicknesses in the dataset are shown in Figure S1 and Figure S2, respectively.

**Figure S1**


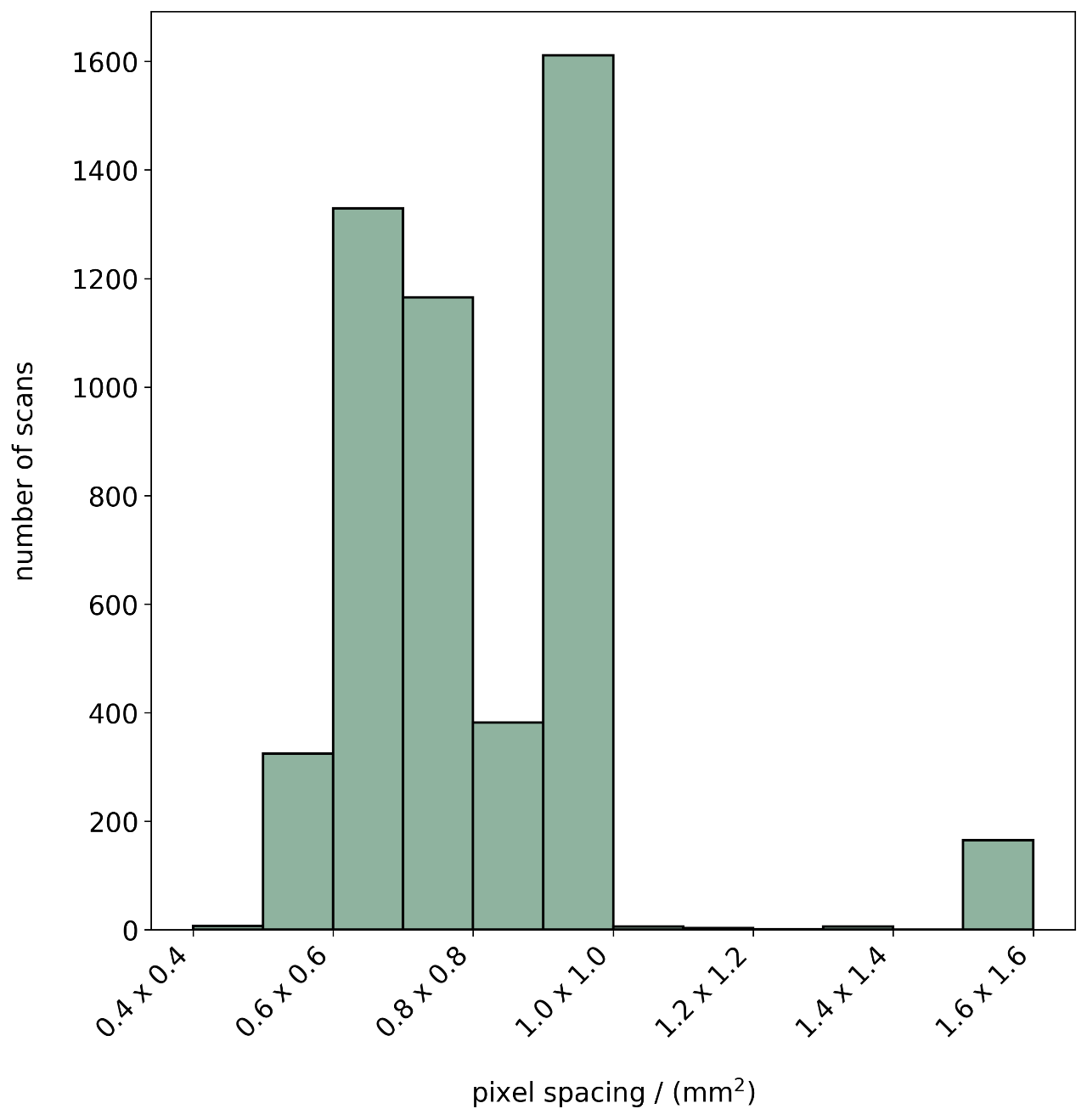


**Figure S1:** Distribution of pixel spacings of the 5000 selected chest CT scans in the dataset.

**Figure S2**


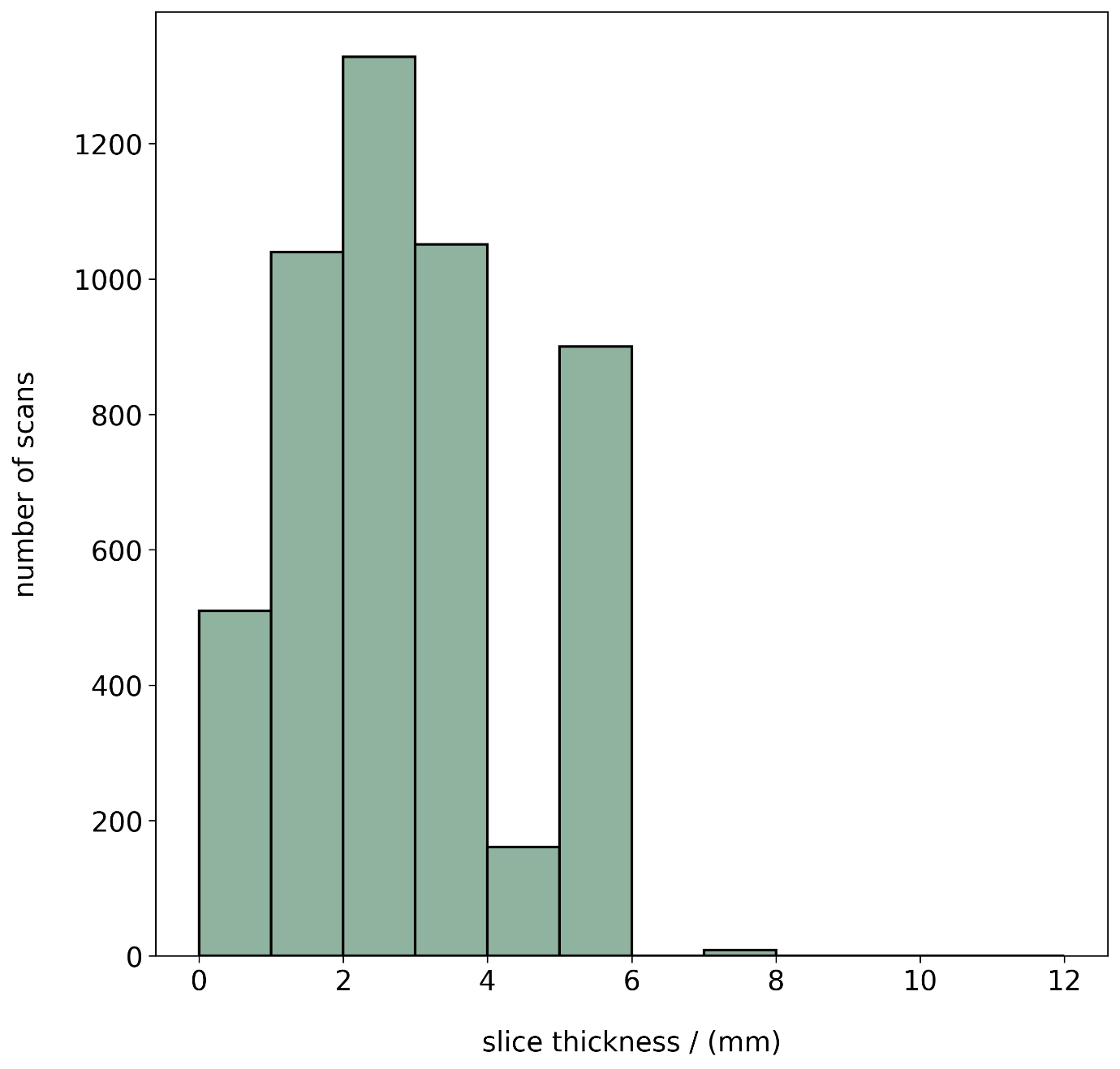


**Figure S2:** Distribution of slice thicknesses of the 5000 selected chest CT scans in the dataset.

## Data preprocessing

The CT scans collected from the PACS were preprocessed before they were used as input for the deep learning model. First, the center between the medial clavicular epiphyseal cartilages was automatically localized in each scan using a dedicated object detection algorithm [[20]](https://www.zotero.org/google-docs/?fiCM2x). Second, all scans were resampled to a voxel spacing of [1.0 mm, 1.0 mm, 1.0 mm] using linear interpolation. Third, a patch of size (112 x 80 x 60) voxels was cropped around the center between the clavicles in each scan. The patch size was derived heuristically and covered the sternoclavicular joints, together with their contributing portions of the sternum and the medial clavicles. Fourth, a CT window with a width of 1000 Hounsfield Units (HU) and a level of 500 HU was applied. This windowing enabled contrast enhancement and highlighted structures we considered relevant for age assessment. Next, all patches were resized to (112 x 112 x 112) voxels using linear interpolation to match the shape of the model input layer. Finally, voxel values were linearly scaled from the value range [-250, 1250] to [0.0, 1.0] for network training.

## Training, validation, and test split

The number of available thoracic CT scans in the PACS decreased with decreasing age at acquisition, resulting in an imbalanced dataset with respect to age. The two data subsets, validation set, and test set, were sampled from the total dataset such that they were balanced in terms of both age and sex. The training set consisted of the remaining data and therefore remained imbalanced. To mitigate this issue, the training data was artificially oversampled during training such that the training batches were balanced on average throughout a training epoch. The age and sex distribution of all datasets are shown in Figure S3.

**Figure S3**

**
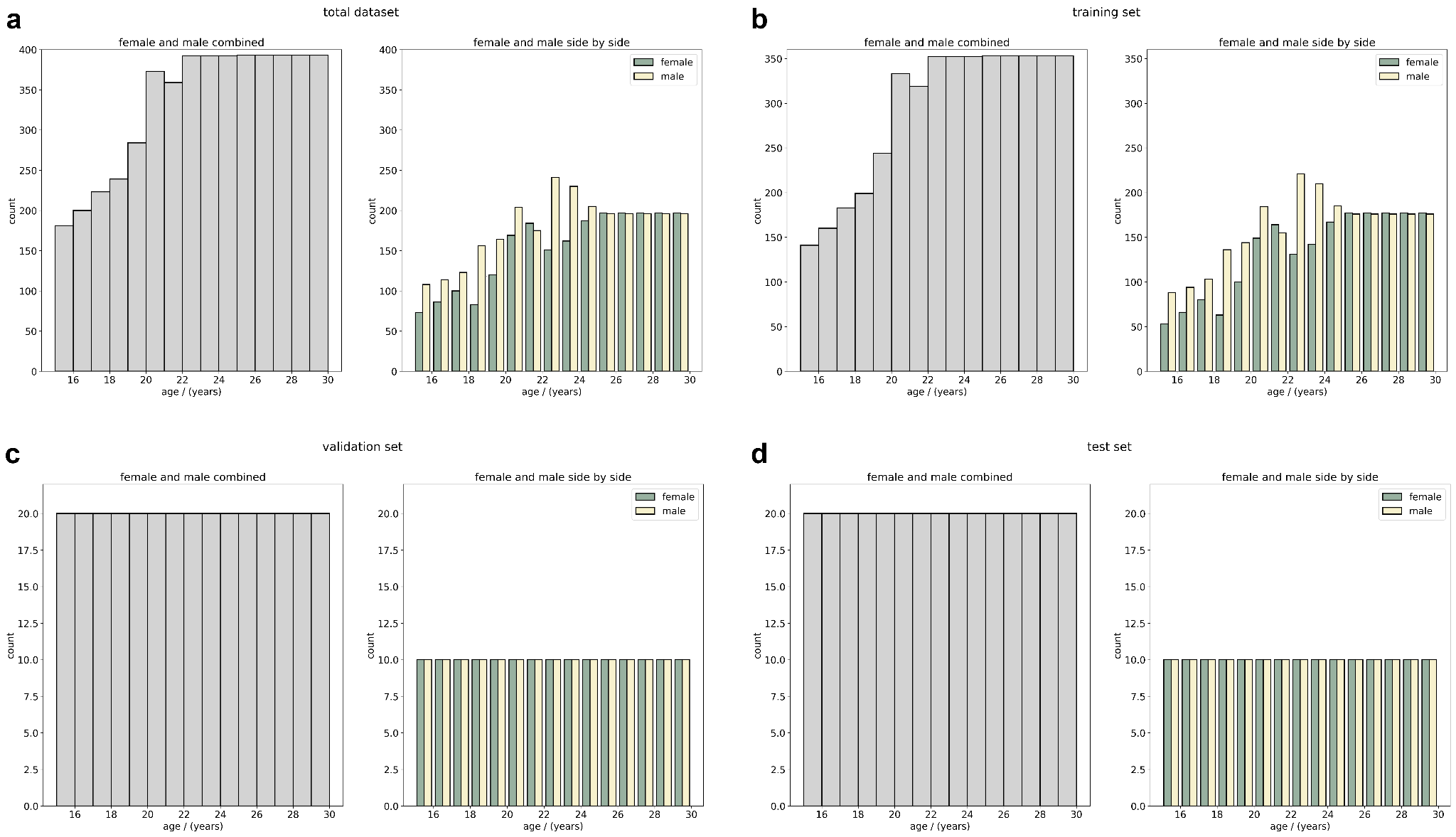
**

**Figure S3:** Age distributions in the data. Subfigure (a) shows the age distribution in the total dataset, which consisted of 5,000 samples from 2,535 patients. The remaining subfigures show the age distribution of the (b) training, (c) validation, and (d) test set. The validation and test were sampled from the total dataset to each have a balanced age and sex distribution, the remaining samples were used as the training set. The histograms have a bin width of 1 year. The left panel in each subfigure displays the combined distribution of both female and male persons in each bin, while the right panel shows the age distribution of female and male persons side-by-side.

## Network architecture and training

The deep learning ensemble model was implemented using PyTorch (version 1.13) [[28]](https://www.zotero.org/google-docs/?WXQT51) and “PyTorch Lightning (version 1.7.7)” (Falcon, W. PyTorch Lightning. <https://www.pytorchlightning.ai> (2020)). The individual ensemble member networks were the same adapted version of the ResNet-18 [[19]](https://www.zotero.org/google-docs/?jB11DT), which processed three-dimensional inputs and fused image data with tabular data. The design choice of fusing the convolutional network’s image embedding (1x496) and the tabular sex information (1x16) through tensor concatenation was motivated by the winning model of the *2017 RSNA Pediatric Bone Age Machine Learning Challenge* [[25]](https://www.zotero.org/google-docs/?uQ2QV0).

For pretraining, a single autoencoder network that shared its architecture with the ensemble member networks in the encoder part was trained to reconstruct 3D patches of raw and not preprocessed CT scans from the training set. The encoder weights of the trained autoencoder were used to initialize the ensemble networks.

Ensemble-member networks were trained separately and, due to the training’s randomness, had different weights that yielded unique predictions for each sample during inference. Each network was trained with the following parameters:

- Loss function $f(x) = x^{2}+x^{3}$
- Batch size = 32
- Optimizer = Adam [[29]](https://www.zotero.org/google-docs/?OAWRHv)
- Learning rate = Gradually decreasing from 0.01 to 0.0001)
- Number of epochs = 400.

The loss function combined a quadratic and cubic loss and was derived heuristically. We observed that it reduced the standard deviation of the absolute prediction error compared to other loss functions, such as mean absolute error or mean squared error. During training, the preprocessed CT scans of the training set were randomly augmented with the following random transformations: flipping, scaling, rotation, translation, and Gaussian noise. Validation and test data were not augmented.

The network complexity of the adapted ResNet-18 is described in Table S1 and the complexity of the original ResNet-18 is described in Table S2 for comparison. Also, both tables include a list of all network layers and their output size for inputs of size (1 x 112 x 112 x 112) and (3 x 112 x 112), respectively. Adding a third dimension to process CT volumes tripled the total number of network parameters from 11,177,025 in the original to 33,401,105 in the adapted ResNet-18.

**Table S1**

| **Layer** | **Output size** | **# Parameters** |
| --- | --- | --- |
| Conv3d: 1-1 | 64 x 56 x 56 x 56 | 8,064 |
| BatchNorm3d: 1-2 | 64 x 56 x 56 x 56 | 128 |
| ReLU: 1-3 | 64 x 56 x 56 x 56 | - |
| MaxPool3d: 1-4 | 64 x 28 x 28 x 28 | - |
| Sequential: 1-5 | 64 x 28 x 28 x 28 | - |
| BuildingBlock_3D: 2-1 | 64 x 28 x 28 x 28 | 221,440 |
| BuildingBlock_3D: 2-2 | 64 x 28 x 28 x 28 | 221,440 |
| Sequential: 1-6 | 128 x 14 x 14 x 14 | - |
| BuildingBlock_3D: 2-3 | 128 x 14 x 14 x 14 | 672,512 |
| BuildingBlock_3D: 2-4 | 128 x 14 x 14 x 14 | 885,248 |
| Sequential: 1-7 | 256 x 7 x 7 x 7 | - |
| BuildingBlock_3D: 2-5 | 256 x 7 x 7 x 7 | 2,688,512 |
| BuildingBlock_3D: 2-6 | 256 x 7 x 7 x 7 | 3,539,968 |
| Sequential: 1-8 | 512 x 4 x 4 x 4 | - |
| BuildingBlock_3D: 2-7 | 512 x 4 x 4 x 4 | 10,750,976 |
| BuildingBlock_3D: 2-8 | 512 x 4 x 4 x 4 | 14,157,824 |
| AdaptiveAvgPool3d: 1-9 | 512 x 1 x 1 x 1 | - |
| Linear: 1-10 | 496 | 254,448 |
| ReLU: 1-11 | 496 | - |
| Linear: 1-12 | 16 | 32 |
| Linear: 1-13 | 1 | 513 |
| Total parameters | - | 33,401,105 |

**Table S1:** Network layer output and parameters of the adapted three-dimensional ResNet-18 [[19]](https://www.zotero.org/google-docs/?HECyoS) used in this study for a monochrome 3D single channel input of shape (1 x 112 x 112 x 112) and an separate input of shape (1 x 1), calculated with the Python package “torchsummary” (Yep, T. torchinfo. *GitHub*. <https://github.com/TylerYep/torchinfo> (2020)).

**Table S2**

| **Layer** | **Output size** | **# Parameters** |
| --- | --- | --- |
| Conv2d: 1-1 | 64 x 56 x 56 | 9,408 |
| BatchNorm2d: 1-2 | 64 x 56 x 56 | 128 |
| ReLU: 1-3 | 64 x 56 x 56 | - |
| MaxPool2d: 1-4 | 64 x 28 x 28 | - |
| Sequential: 1-5 | 64 x 28 x 28 | - |
| BasicBlock: 2-1 | 64 x 28 x 28 | 73,984 |
| BasicBlock: 2-2 | 64 x 28 x 28 | 73,984 |
| Sequential: 1-6 | 128 x 14 x 14 | - |
| BasicBlock: 2-3 | 128 x 14 x 14 | 230,144 |
| BasicBlock: 2-4 | 128 x 14 x 14 | 295,424 |
| Sequential: 1-7 | 256 x 7 x 7 | - |
| BasicBlock: 2-5 | 256 x 7 x 7 | 919,040 |
| BasicBlock: 2-6 | 256 x 7 x 7 | 1,180,672 |
| Sequential: 1-8 | 512 x 4 x 4 | - |
| BasicBlock: 2-7 | 512 x 4 x 4 | 3,673,088 |
| BasicBlock: 2-8 | 512 x 4 x 4 | 4,720,640 |
| AdaptiveAvgPool2d: 1-9 | 512 x 1 x 1 | - |
| Linear: 1-10 | 1 | 513 |
| Total parameters | - | 11,177,025 |

**Table S2**: Network layer output and parameters of a PyTorch ResNet-18 [[19, 28]](https://www.zotero.org/google-docs/?UXLav6) for a 2D RGBinput with three color channels of shape (3 x 112 x 112), calculated with the Python package “torchsummary” (Yep, T. torchinfo. GitHub. <https://github.com/TylerYep/torchinfo> (2020)).

## Binary classification for the age threshold of 18.0 years

In a separate analysis we evaluated the binary classification performance of both approaches (deep learning and HRE) for predicting whether a person is >= 18 years or < 18 years. To this end, deep learning predictions were categorized as >= 18 years or < 18 years. For the optimistic human reader performance estimate, predictions were derived by first assigning a single ossification stage to each person in the test set using weighted random sampling. The weights for each stage were set to $p_{s}\left( x \right)$ (see Methods for calculation). Next, the mean age of the sampled stage was used as the predicted age. Afterwards, the predictions were also categorized as >= 18 years or < 18 years. Prediction performance was evaluated using binary accuracy, sensitivity, and specificity.

The categorized deep learning predictions had a binary accuracy of 0.89, sensitivity of 0.97 and specificity of 0.5. Categorized predictions derived from the optimistic human reader performance estimate had a binary accuracy of 0.91, sensitivity of 0.95 and specificity of 0.73.
